# Supplementary figures and images for: Inequalities in children’s mental health care: analysis of routinely collected data on prescribing and referrals to secondary care
Source: BMC Psychiatry. 2023 Jan 11;23:22. doi: 10.1186/s12888-022-04438-5 (PMC9831880; doi:10.1186/s12888-022-04438-5)

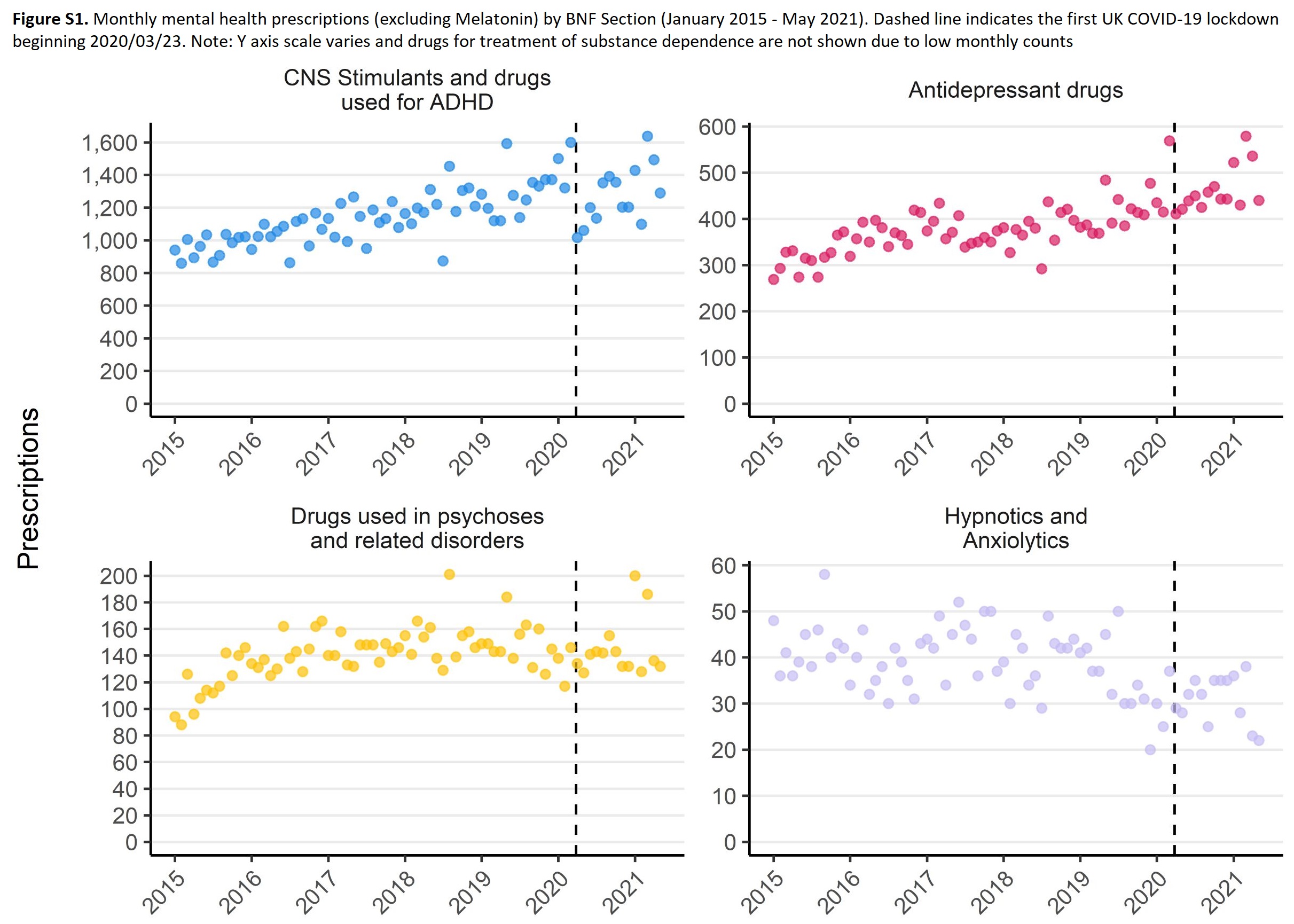

Supplement: Supplementary file 1 — Additional file 1. [file 12888_2022_4438_MOESM1_ESM.jpg]

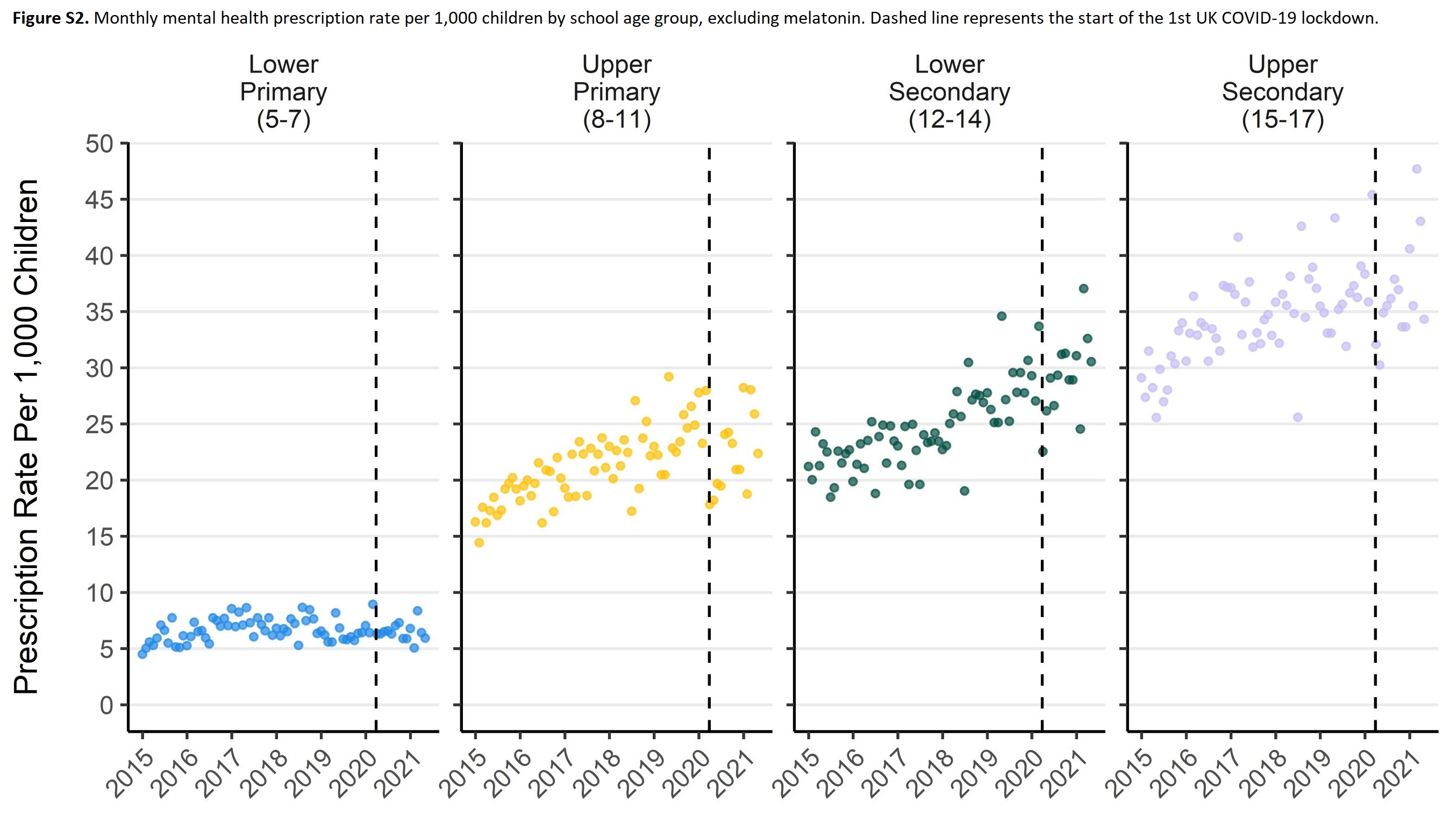

Supplement: Supplementary file 2 — Additional file 2. [file 12888_2022_4438_MOESM2_ESM.jpg]

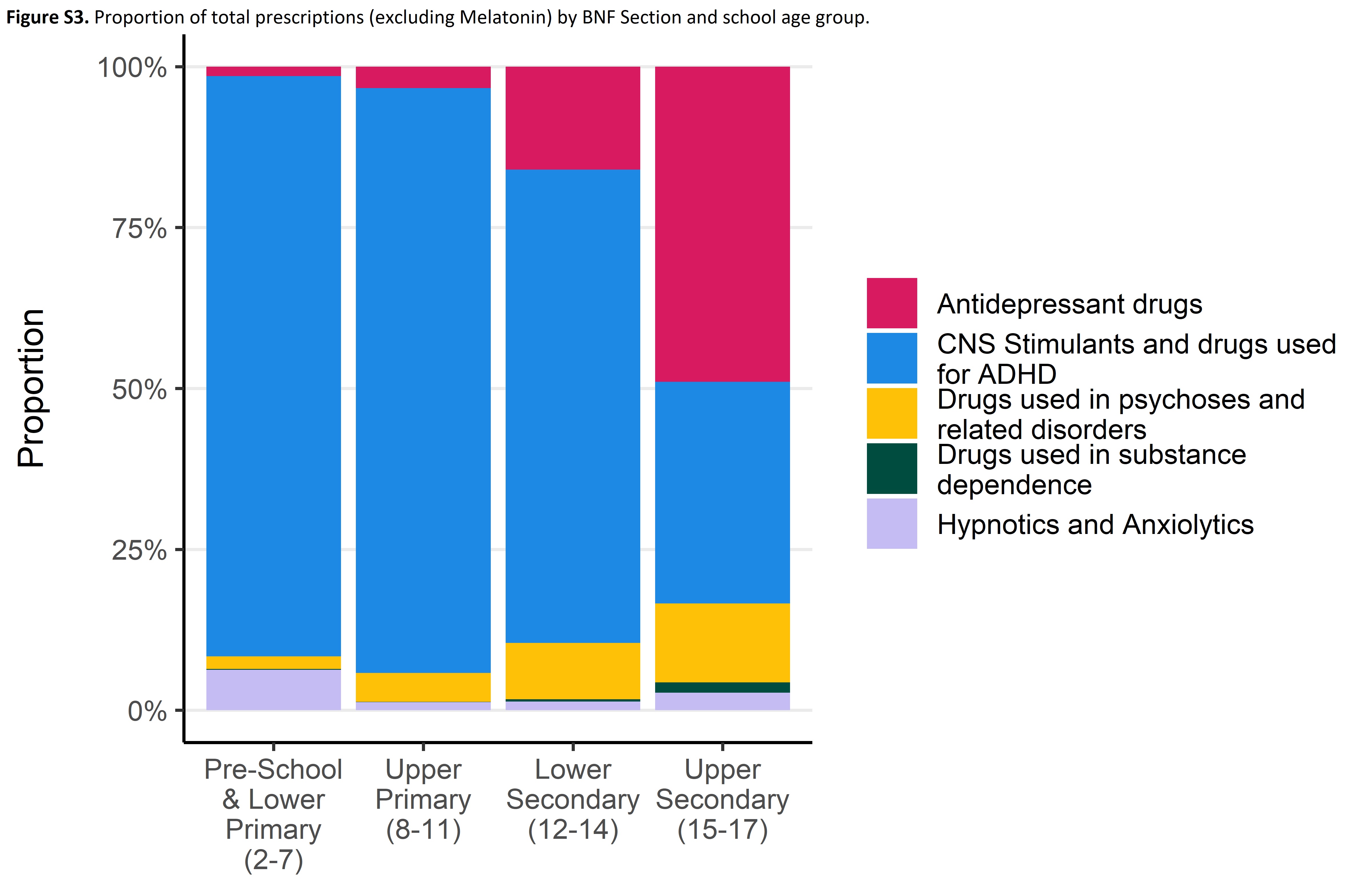

Supplement: Supplementary file 3 — Additional file 3. [file 12888_2022_4438_MOESM3_ESM.jpg]

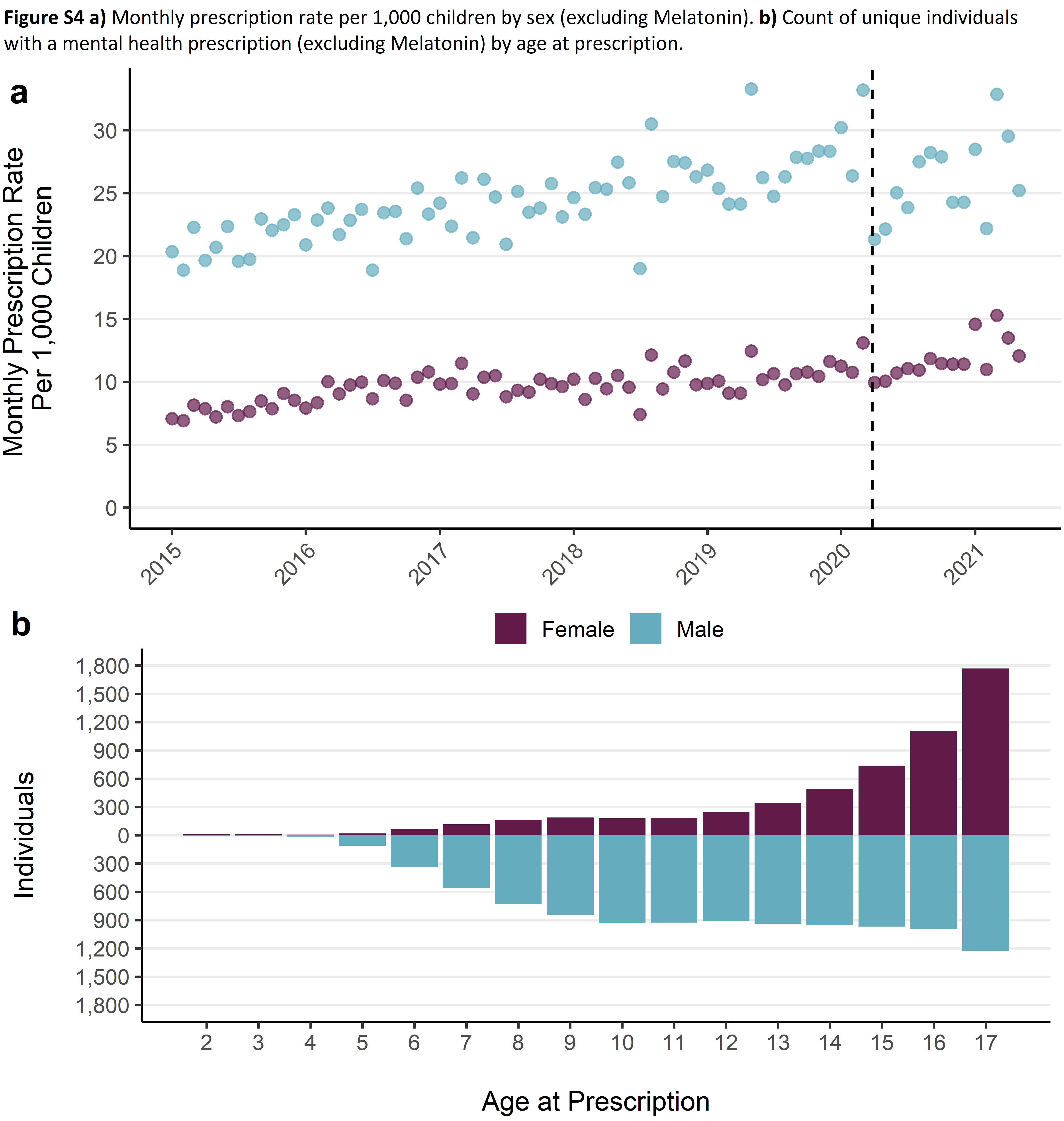

Supplement: Supplementary file 4 — Additional file 4. [file 12888_2022_4438_MOESM4_ESM.jpg]

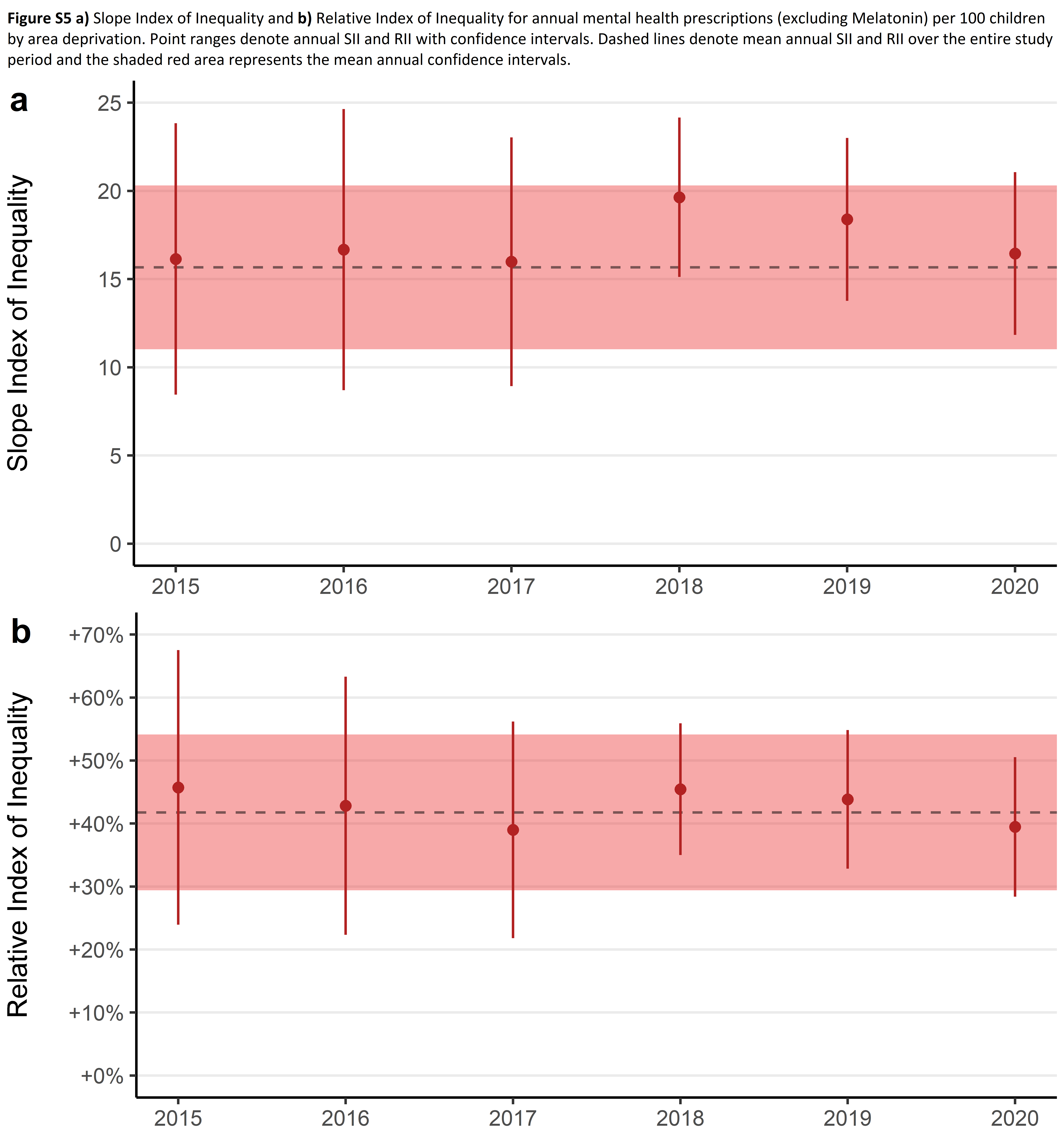

Supplement: Supplementary file 5 — Additional file 5. [file 12888_2022_4438_MOESM5_ESM.jpg]
