# Supplementary material for: Inequalities in children’s mental health care: analysis of routinely collected data on prescribing and referrals to secondary care
Source: BMC Psychiatry. 2023 Jan 11;23:22. doi: 10.1186/s12888-022-04438-5 (PMC9831880; doi:10.1186/s12888-022-04438-5)
Supplement: Supplementary file 7 — Additional file 7: Table S2. Credit taxonomy for author contributions. [file 12888_2022_4438_MOESM7_ESM.pdf]

Table S2. Credit taxonomy for author contributions.

Term	Description	Authors
Conceptualization	https://credit.niso.org/contributor-roles/conceptualization/	All authors
Data curation	https://credit.niso.org/contributor-roles/data-curation/	HR, WB, JB, KW on behalf of Grampian DaSH
Formal analysis	https://credit.niso.org/contributor-roles/formal-analysis/	WB, JB
Funding acquisition	https://credit.niso.org/contributor-roles/funding-acquisition/	JB, CB, SG, KW
Investigation	https://credit.niso.org/contributor-roles/investigation/	WB, JB
Methodology	https://credit.niso.org/contributor-roles/methodology/	WB, JB, CB, SP,
Project administration	https://credit.niso.org/contributor-roles/project-administration/	JB, CB, SP, KW
Resources	https://credit.niso.org/contributor-roles/resources/	DR, ET, KW, HR
Software	https://credit.niso.org/contributor-roles/software/	WB, JB
Supervision	https://credit.niso.org/contributor-roles/supervision/	JB, CB, SP
Validation	https://credit.niso.org/contributor-roles/validation/	JB, WB
Visualization	https://credit.niso.org/contributor-roles/visualization/	WB, JB
Writing – original draft	https://credit.niso.org/contributor-roles/writing-original-draft/	WB
Writing – review & editing	https://credit.niso.org/contributor-roles/writing-review-editing/	All authors
Patient and Public Engagement		MR, SG, BO, WB, JB
