# Supplementary material for: Inequalities in children’s mental health care: analysis of routinely collected data on prescribing and referrals to secondary care
Source: BMC Psychiatry. 2023 Jan 11;23:22. doi: 10.1186/s12888-022-04438-5 (PMC9831880; doi:10.1186/s12888-022-04438-5)
Supplement: Supplementary file 8 — Additional file 8: Table S3. List of mental health medications by British National Formulary Section and Subsection. [file 12888_2022_4438_MOESM8_ESM.pdf]

Table S3. List of mental health medications by British National Formulary Section and Subsection

BNF Section Code	Section Name	BNF Subsection Code	Subsection Name	Approved Name
401	**Hypnotics and Anxiolytics**	40101	***Hypnotics***	Melatonin<br>Zopiclone<br>Temazepam<br>Nitrazepam<br>Zolpidem<br>Chloral Hydrate<br>Cloral Betaine<br>Sodium Oxybate
		40102	***Anxiolytics***	Diazepam<br>Lorazepam<br>Buspirone Hydrochloride
402	**Drugs used in psychoses and related disorders**	40201	***Antipsychotic drugs***	Olanzapine<br>Risperidone<br>Aripiprazole<br>Chlorpromazine Hydrochloride<br>Quetiapine<br>Haloperidol<br>Sulpiride<br>Levomepromazine<br>Trifluoperazine<br>Promazine Hydrochloride<br>Lurasidone Hydrochloride
		40202	***Antipsychotic depot injections***	Aripiprazole
		40203	***Drugs used for mania and hypomania***	Lithium Carbonate<br>Sodium Valproate
403	**Antidepressant drugs**	40301	***Tricyclic and related antidepressant drugs***	Amitriptyline<br>Nortriptyline<br>Clomipramine Hydrochloride<br>Imipramine Hydrochloride<br>Lofepramine<br>Trazodone Hydrochloride<br>Dosulepin Hydrochloride
		40302	***Monoamine-oxidase inhibitors***	Phenelzine
		40303	***Selective serotonin re-uptake inhibitors***	Sertraline<br>Fluoxetine<br>Citalopram<br>Escitalopram<br>Fluvoxamine Maleate<br>Paroxetine
		40304	***Other antidepressant drugs***	Mirtazapine<br>Duloxetine<br>Flupentixol<br>Venlafaxine<br>Agomelatine<br>Vortioxetine Hydrobromide
404	**CNS Stimulants and drugs used for ADHD**	40400	***CNS Stimulants and drugs used for ADHD***	Methylphenidate Hydrochloride<br>Atomoxetine<br>Lisdexamfetamine Dimesylate<br>Dexamfetamine Sulfate<br>Modafinil<br>Guanfacine Hydrochloride
410	**Drugs used in substance dependence**	41001	***Alcohol dependence***	Acamprosate Calcium
		41002	***Nicotine dependence***	Nicotine<br>Varenicline Tartrate
		41003	***Opioid dependence***	Methadone Hydrochloride<br>Buprenorphine And Naloxone
